# A recombinant *Fasciola gigantica* 14-3-3 epsilon protein (rFg14-3-3e) modulates various functions of goat peripheral blood mononuclear cells

**DOI:** 10.1186/s13071-018-2745-4

**Published:** 2018-03-06

**Authors:** Ai-Ling Tian, MingMin Lu, Guillermo Calderón-Mantilla, Evangelia Petsalaki, Tania Dottorini, XiaoWei Tian, YuJian Wang, Si-Yang Huang, Jun-Ling Hou, XiangRui Li, Hany M. Elsheikha, Xing-Quan Zhu

**Affiliations:** 1 State Key Laboratory of Veterinary Etiological Biology, Key Laboratory of Veterinary Parasitology of Gansu Province, Lanzhou Veterinary Research Institute, Chinese Academy of Agricultural Sciences, Lanzhou, Gansu Province 730046 People’s Republic of China; 2 College of Veterinary Medicine, Nanjing Agricultural University, Nanjing, 210095 People’s Republic of China; 3 European Molecular Biology Laboratory-European Bioinformatics Institute, Wellcome Genome Campus, Hinxton, CB10 1SD UK; 4 Faculty of Medicine and Health Sciences, School of Veterinary Medicine and Science, University of Nottingham, Sutton Bonington Campus, Loughborough, LE12 5RD UK; 5 Jiangsu Co-innovation Center for the Prevention and Control of Important Animal Infectious Diseases and Zoonoses, Yangzhou University College of Veterinary Medicine, Yangzhou, Jiangsu Province 225009 People’s Republic of China

**Keywords:** *Fasciola gigantica*, 14-3-3 epsilon protein isoform, Homology modelling, Goat, Peripheral blood mononuclear cells

## Abstract

**Background:**

The molecular structure of *Fasciola gigantica* 14-3-3 protein has been characterized. However, the involvement of this protein in parasite pathogenesis remains elusive and its effect on the functions of innate immune cells is unknown. We report on the cloning and expression of a recombinant *F. gigantica* 14-3-3 epsilon protein (rFg14-3-3e), and testing its effects on specific functions of goat peripheral blood mononuclear cells (PBMCs).

**Methods:**

rFg14-3-3e protein was expressed in *Pichia pastoris*. Western blot and immunofluorescence assay (IFA) were used to examine the reactivity of rFg14-3-3e protein to anti-*F. gigantica* and anti-rFg14-3-3e antibodies, respectively. Various assays were used to investigate the stimulatory effects of the purified rFg14-3-3e protein on specific functions of goat PBMCs, including cytokine secretion, proliferation, migration, nitric oxide (NO) production, phagocytosis, and apoptotic capabilities. Potential protein interactors of rFg14-3-3e were identified by querying the databases Intact, String, BioPlex and BioGrid. A Total Energy analysis of each of the identified interaction was performed. Gene Ontology (GO) enrichment analysis was conducted using Funcassociate 3.0.

**Results:**

Sequence analysis revealed that rFg14-3-3e protein had 100% identity to 14-3-3 protein from *Fasciola hepatica*. Western blot analysis showed that rFg14-3-3e protein is recognized by sera from goats experimentally infected with *F. gigantica* and immunofluorescence staining using rat anti-rFg14-3-3e antibodies demonstrated the specific binding of rFg14-3-3e protein to the surface of goat PBMCs. rFg14-3-3e protein stimulated goat PBMCs to produce interleukin-10 (IL-10) and transforming growth factor beta (TGF-β), corresponding with low levels of IL-4 and interferon gamma (IFN-γ). Also, this recombinant protein promoted the release of NO and cell apoptosis, and inhibited the proliferation and migration of goat PBMCs and suppressed monocyte phagocytosis. Homology modelling revealed 65% identity between rFg14-3-3e and human 14-3-3 protein YWHAE. GO enrichment analysis of the interacting proteins identified terms related to apoptosis, protein binding, locomotion, hippo signalling and leukocyte and lymphocyte differentiation, supporting the experimental findings.

**Conclusions:**

Our data suggest that rFg14-3-3e protein can influence various cellular and immunological functions of goat PBMCs *in vitro* and may be involved in mediating *F. gigantica* pathogenesis. Because of its involvement in *F. gigantica* recognition by innate immune cells, rFg14-3-3e protein may have applications for development of diagnostics and therapeutic interventions.

**Electronic supplementary material:**

The online version of this article (10.1186/s13071-018-2745-4) contains supplementary material, which is available to authorized users.

## Background

*Fasciola gigantica* is a worldwide distributed liver fluke that can infect a wide range of livestock of economic importance such as cattle, sheep and goats [1]. Infections with *Fasciola* spp. result in reduced meat, milk and wool production [2], with an estimated economic loss more than 3 billion USD per annum worldwide [3, 4]. In addition, disease caused by *Fasciola* infection (known as fasciolosis) in humans is included in the WHO top listed neglected tropical diseases [5, 6], and it is estimated that at least 2.4 million people are infected worldwide and 180 million are at risk of infections [2].

The mammalian definitive hosts of *F. gigantica* become infected by ingestion of vegetation containing encysted metacercariae. Infective metacercariae emerge in the duodenum, rapidly traverse the intestinal wall, and migrate through the peritoneal cavity to reach the liver capsule. Following a period of burrowing, feeding and growth within the liver parenchyma, the flukes reach their final destination within the bile ducts where they attain their sexual maturity and produce eggs [7, 8]. During their migration and development, liver flukes encounter various host tissue types, distinct physiological microenvironments, and a combination of both humoral and cellular immune responses. *F. gigantica* can remarkably tackle these challenges and survive for extended periods inside its host [9].

Although host immune evasion is a common strategy used by successful parasites, *Fasciola* spp. provoke recognition by host immune cells less capable of destroying them. To accomplish this, they use virulence-associated excretory-secretory products (ESPs) to manipulate cellular processes [10], such as immune evasion and immune subversion [11, 12] to promote parasite survival. Among the virulence factors in the ESPs that are involved in host-parasite interaction, the 14-3-3 proteins are of interests given their ability to bind to many signaling proteins, such as kinases and phosphatases, and to mediate many regulatory processes, metabolism and signal transduction pathways [13, 14]. The 14-3-3 family encompasses a group of conserved intracellular polypeptides with molecular weight of 28–33 kDa, which are found in all eukaryotic organisms [15]. These proteins have been the focus of much research due to their roles in modulating various protein-protein interactions [16, 17].

Various structural and functional aspects of 14-3-3 protein have been studied in *Toxoplasma gondii* [18], *Schistosoma japonicum* [19], *Eimeria tenella* [20], *Echinococcus granulosus* [21], *Haemonchus contortus* [22] and *Trichinella spiralis* [23], and the putative role of 14-3-3 protein in parasite pathogenesis has been reported [24–27]. However, the biological effects of *F. gigantica* 14-3-3 protein on the host innate immune system are still largely unknown.

In the present study, the gene encoding *F. gigantica* 14-3-3 epsilon protein (Fg14-3-3e) isoform was cloned and expressed in *Pichia pastoris*. The modulatory effects of the purified rFg14-3-3e on various functions of the goat peripheral blood mononuclear cells (PBMCs), including cytokine secretion, proliferation, migration, nitric oxide (NO) production, phagocytosis and apoptosis were studied using a range of assays. These experimental studies were complemented by *in silico* homology modelling analysis. Our data suggest that rFg14-3-3e protein can significantly influence the cellular and immunological functions of goat PBMCs, all critical pieces of the immunopathogenesis of *F. gigantica* infection.

## Methods

### Animals used in the experiments

Local, 3- to 6-month-old crossbred goats were obtained from the teaching and research flock at Nanjing Agricultural University. Goats were fed with hay and whole shelled corn, and were housed indoor in pens and provided with water *ad libitum*. All goats were treated with triclabendazole (50 mg/kg body weight) in order to eliminate any potential previous infection with *Fasciola* spp. A fecal sample from each goat was microscopically examined for helminth eggs 2 weeks after treatment. Only goats demonstrating no eggs were included in the study and daily health checks were performed throughout the experiment. Three biological replicates (three goats), each consisting of three technical replicates (i.e. three samples from each goat), were used in this study. For the production of antibodies, female Sprague Dawley (SD) rats (body weight ~150 g) were purchased from the Experimental Animal Center of Jiangsu, PR China (Qualified Certificate: SCXK 2008-0004) and were housed under specific-pathogen-free conditions in the lab animal facility and fed sterilized food and water *ad libitum*. All efforts were made to minimize suffering of animals.

### Isolation of goat PBMCs

Goat peripheral venous blood samples were collected from healthy goats. The goat PBMCs were isolated from freshly collected blood samples using commercially available goat PBMCs’ isolation kit (TBD, Tianjin, China). The number of goat PBMCs were adjusted to a density of 1 × 10^6^ cells/ml in RPMI 1640 medium containing 10% fetal bovine serum (FBS), 1% penicillin-streptomycin (Gibco, New York, USA). Goat PBMCs were maintained in a humidified atmosphere of 5% CO_2_ at 37 °C. Cells viability was determined by trypan blue dye exclusion, and only cells with more than 95% viability were used in the experiments. Monocytes were isolated from goat PBMCs based on their tendency to adhere to the plastic surface of the cell culture flasks [28].

### Parasite strain

Adult flukes of *F. gigantica* were collected from the gall-bladder of naturally infected buffaloes slaughtered for human consumption at local abattoirs in Guangxi Zhuang Autonomous Region, PR China. After washing in phosphate buffered saline (PBS, pH 7.4), the flukes were collected and immediately used for RNA isolation or stored at -80 °C with RNA stabilizer until use. The species identity was confirmed as *F. gigantica* based on PCR and sequencing of the second internal transcribed spacer (ITS2) of ribosomal DNA [29], which yielded absolute homology to ITS2 sequence of *F. gigantica* from Guangxi Zhuang Autonomous Region (GenBank: AJ557569).

### Recombinant plasmid construction and sequencing of *Fg14-3-3e* gene

Due to the limited genomic data on *F. gigantica*, we searched *Fasciola hepatica* ESPs dataset acquired by liquid chromatography-tandem mass spectrometry (LC-MS/MS) and *F. hepatica* cDNA library from previous proteomic studies to screen for homologous 14-3-3 protein sequences using the BLAST program (https://blast.ncbi.nlm.nih.gov/Blast.cgi). This analysis identified *F. hepatica 14-3-3 epsilon protein isoform* (*Fh14-3-3e*) sequence, which was used to design primers to amplify *F. gigantica 14-3-3 epsilon protein isoform* (*Fg14-3-3e*) sequence. First-strand cDNA was synthesized from total *F*. *gigantica* RNA, isolated from 30 mg of adult *F*. *gigantica* flukes using Trizol reagent (Invitrogen, San Diego, USA), by reverse transcription polymerase chain reaction (RT-PCR) using RevertAid First Strand cDNA Synthesis Kit (Thermo Scientific (EU), Vilnius, Lithuania). The cDNA was used for amplification of *Fg14-3-3e* gene using a forward primer (5'-CCG GAA TTC GAG GAG GTG AAT ATC G-3') and a reverse primer (5'-ATT T G CGG CCG CCT AAG CCT TTT TCT CCT C-3'). These specific primers of *Fg14-3-3e* gene were designed based on the published sequence of *Fh14-3-3e* gene (GenBank: LN629113.1). *Eco*RI and *Not*I restriction sites, indicated by underline, were incorporated into the primers to facilitate cloning. The amplified *Fg14-3-3e* gene was digested with *Eco*RI and *Not*I and spliced into the corresponding cloning sites in the plasmid vector pMD19-T (Takara, Dalian, Liaoning, China). The recombinant plasmid was transformed into *Trans5α* chemically competent cells (TransGen Biotech, Beijing, China). Several positive clones were selected and sequenced by GenScript (Nanjing, Jiangsu, China) to confirm the correct insertion/orientation of *Fg14-3-3e* gene in the vector.

### Characterization of the *Fg14-3-3e* gene sequence

We characterized the *Fg14-3-3e* gene sequence by translating the cDNA sequence into the amino acid sequences using Seaview [30]. Then, three different approaches were used to identify and annotate signatures of functional mechanisms present in the amino acidic sequence. Specifically, the protein sequence was first subjected to the prediction of sequence domain, followed by comparative analysis and 3D modelling. The presence of sequence domain was performed using SMART (Simple Modular Architecture Research Tool) [31]. Next, a comparative analysis was done to search for similarities in protein sequence databases (NCBI non-redundant protein sequence) and to identify possible homologous proteins. Furthermore, we predicted the three-dimensional model of the homo-dimer using homology modelling (Swissmodel) [32]. The signal peptide was predicted by SignalP 4.1 Server (http://www.cbs.dtu.dk/services/SignalP/), the transmembrane region was predicted by TMHMM Server v. 2.0 (http://www.cbs.dtu.dk/services/TMHMM/) and the potential N-glycosylation sites were predicted by NetNGlyc 1.0 Server (http://www.cbs.dtu.dk/services/NetNGlyc/).

### Expression of rFg14-3-3e protein

Positive clones, containing the *Fg14-3-3e* gene in the correct orientation were selected. The purified *Fg14-3-3e* gene fragment was sub-cloned into pPIC9K vector. The constructs were designed with appropriate restriction sites and a carboxyl-terminal His6 tag to enable purification. The plasmid pPIC9K-*Fg14-3-3e* was linearized with *Sal*I to transform the Methylotrophic Yeast *P*. *pastoris* GS115 strain by electroporation using a GenePulser XcellTM (Bio-Rad, Hercules, California, USA). Positive recombinant *P. pastoris* clones containing the insert were selected for expression by inoculating into 15 ml of buffered complex medium containing glycerol (BMGY). The inoculated BMGY medium [1% (wt/vol) yeast extract, 2% (wt/vol) peptone, 1% (wt/vol) yeast nitrogen base, 1% (wt/vol) glycerol, 0.00004% (wt/vol) biotin, and 0.1 M potassium phosphate (pH 6.0)] in 100 ml conical flasks was grown at 28 °C with vigorous shaking for 24 h. The cells were harvested by centrifugation (250× *g* for 10 min), resuspended in 20 ml of buffered complex medium containing methanol (BMMY; BMGY medium with 1% methanol substituted for glycerol). The culture was allowed to continue growing for four days. During this *Fg14-3-3e* gene expression induction period, methanol was added every 24 h to maintain a final concentration of 1% (v/v). The cells were then pelleted by centrifugation (2,500× *g* at 4 °C for 10 min) and the culture supernatant was harvested for protein extraction. *P. pastoris* cells transformed with vector pPIC9K (without insert) were similarly treated and analyzed as a negative control.

### Protein extraction and SDS-PAGE

Following extraction of the total protein from the yeast culture supernatant by precipitation with 10% trichloroacetic acid, the protein pellet was washed several times with acetone and finally dissolved in distilled water. The size of the isolated rFg14-3-3e protein was analyzed in 12% sodium dodecyl sulfate-polyacrylamide gel electrophoresis (SDS-PAGE) gels, followed by Coomassie blue staining, and the concentration of the protein was determined using the Bradford method, using bovine serum albumin (BSA) as the standard. Purified proteins were stored frozen in 1× PBS at -80 °C until further analysis.

### Preparation of polyclonal antibodies

Five goats, raised in helminth-free conditions, were infected orally with 500 viable encysted metacercariae per animal. One month later, the goat antisera were collected and stored frozen in aliquots at -80 °C until use in Western blot analysis. To generate specific antibodies against rFg14-3-3e protein, 300 μg of the purified protein mixed with complete Freund’s adjuvant (1:1) were injected subcutaneously into SD rats for primary immunization. SD rats were boosted four times with the same dose of the recombinant protein in incomplete Freund’s adjuvant at 2-week intervals. One week after the last injection, serum containing specific anti-rFg14-3-3e antibodies was collected and stored frozen at -80 °C for later use in immunofluorescence visualization of rFg14-3-3e protein binding to the surface of the goat PBMCs.

### Western blotting

Extracted rFg14-3-3e protein samples (20 μg) were resolved on 12% SDS-PAGE gels and transferred onto Hybond-C extra nitrocellulose membrane (Amersham, London, UK). Non-specific binding sites were blocked by immersing the membranes in 5% skim (non-fat) milk in Tris-buffered saline containing 0.1% Tween-20 (TBST) for 1 h at ambient temperature. The membranes were then washed 5 times (5 min each) in TBST. Subsequently, the membranes were incubated with the primary antibodies (antiserum from goats experimentally infected with *F*. *gigantica*) for 1 h at 37 °C (1:100 in TBST). After being washed 5 times with TBST, the membranes were incubated with HRP-conjugated rabbit anti-goat IgG (Sigma, St. Louis, MO, USA) for 1 h at 37 °C (1:2000 in TBST). Finally, the immunoreaction was visualized using freshly prepared 3,3′-diaminobenzidine (DAB, Sigma) as a chromogenic substrate after 5 min.

### Immunofluorescence staining of rFg14-3-3e protein binding to goat PBMCs

Freshly isolated goat PBMCs were incubated with rFg14-3-3e protein for 1 h in a humidified atmosphere of 5% CO_2_ at 37 °C. To minimize background staining, goat PBMCs were fixed with 4% paraformaldehyde, washed 3 times in PBS (5 min each) and were treated with blocking solution (4% BSA in PBS) for 1 h at ambient temperature. rFg14-3-3e-treated or non-treated control goat PBMCs were incubated with rat anti-rFg14-3-3e antibody (1:100) for 1 h at 37 °C, followed by staining with Cy3 conjugated goat anti-rat IgG secondary antibody (1:500) (Beyotime, Haimen, Jiangsu, China) for 1 h at 37°C. Hoechst 33342 (Invitrogen, Eugene, Oregon, USA) was used for nuclear staining. Stained cells were imaged with 100× magnification using a Zeiss laser scanning confocal microscope (LSM710, Zeiss, Jena, Germany) and digital images were analyzed by Zen 2012 imaging software.

### ELISA analysis of the levels of cytokines

Goat PBMCs were seeded into a 24-well tissue culture plate at 10^6^ cells/well in 1 ml medium consisting of RPMI 1640 medium. Serial concentrations (10 μg/ml, 20 μg/ml, 40 μg/ml and 80 μg/ml) of rFg14-3-3e protein were added to the wells. The sham-treated PBMCs served as controls, and the plate was incubated in a humidified atmosphere of 5% CO_2_ at 37 °C for 24 h. The supernatants were collected, and assayed for cytokine production using the goat enzyme linked immunosorbent assay (ELISA) kits (Mlbio, Shanghai, China). Goat PBMCs supernatants were assayed for the production of interleukin-4 (IL-4), IL-10, interferon gamma (IFN-γ), and transforming growth factor beta (TGF-β). Limits of detection were between 2 and 800 pg/ml depending on the analytic assay.

### Inhibitory effect of rFg14-3-3e protein on cell proliferation

Anti-proliferative effects of rFg14-3-3e protein on goat PBMCs were determined by performing Cell Counting Kit-8 (CCK-8) assay (Beyotime, Haimen, Jiangsu, China). Goat PBMCs were seeded on 96-well tissue culture plates (10^4^ cells /well in 100 μl), with three replicates used for each rFg14-3-3e protein concentration (10 μg/ml, 20 μg/ml, 40 μg/ml, and 80 μg/ml). Cell cultured plates were incubated at 37 °C in a humidified atmosphere with 5% CO_2_. Following 72 h incubation, 10 μl of CCK-8 reagent was added to each well and the cultures were further incubated at 37 °C in a humidified atmosphere of 5% CO_2_ at 37 °C for 4 h in the dark. The optical density at 450 nm (OD450) was measured using a microplate reader (Bio-Rad).

### *In vitro* cell migration assay

Cell migration assay was performed using 6.5 mm Transwell® with 8.0 μm-pore-size polyester membrane inserts (Corning, Kennebunk, USA). Aliquots of 10^6^ goat PBMCs in 1 ml RPMI culture medium were placed into the top chamber of the transwell in a 24-well tissue culture plate. Cells were stimulated with serial concentrations of rFg14-3-3e protein and incubated in a humidified atmosphere of 5% CO_2_ at 37 °C for 24 h. After incubation, the cells were collected from the bottom chamber and the number of cells that migrated through the membrane into the lower chamber was determined using Automated Cell Counter (Countstar, Shanghai, China). The results were presented as percentages of the seeded goat PBMCs.

### Generation of nitric oxide

Nitric oxide production by goat PBMCs was evaluated by measuring intracellular nitrite by Griess reaction following the protocol of Total Nitric Oxide Assay Kit (Beyotime, Haimen, Jiangsu, China). Briefly, goat PBMCs were seeded at 10^6^ cells/well into a 24-well tissue culture plate. Cells were incubated for 24 h with various concentrations of rFg14-3-3e protein in RPMI 1640 medium at 37 °C in a humidified atmosphere with 5% CO_2_. Absorbance values of the colored solution were measured using a plate microplate reader (Bio-Rad) at 540 nm. NO levels were calculated against a standard curve generated using 0 to 80 μM/l sodium nitrites.

### FITC-dextran uptake

The phagocytic activity of goat monocytes was assessed by measuring the uptake of fluorescein isothiocyanate (FITC)-dextran according to a previously described method [33]. Briefly, freshly isolated goat monocytes were seeded at 10^6^ cells/well into a 24-well tissue culture plate. Goat monocytes were incubated for 48 h with various concentrations of rFg14-3-3e protein in RPMI 1640 medium in a humidified atmosphere of 5% CO_2_ at 37 °C. Then, goat monocytes were re-suspended in 100 μl of cold PBS, and incubated with 1 mg/ml FITC-dextran (average molecular weight of dextran, 59,000 to 77,000; Sigma-Aldrich) in RPMI 1640 medium at 4 or 37 °C for 1 h. After incubation, chilled PBS containing 2% FBS was added to halt phagocytosis and then cells were washed (three times with ice-cold PBS) and resuspended in ice-cold PBS containing 2% paraformaldehyde. FITC-dextran internalization of monocytes was analyzed by flow cytometry (BD Biosciences, San Jose, California, USA). Data were analyzed using FlowJo 10.1 software (Tree Star, Ashland, Oregon, USA). Goat monocytes FITC-dextran (phagocytosis index) was calculated as the change in Median Fluorescence Intensity (MFI) between cell samples incubated at 37 °C and 4 °C.

### Apoptosis assay using Annexin V/PI staining

Apoptosis in goat PBMCs was evaluated and quantified by flow cytometry (BD Biosciences) using the Annexin V-FITC kit (Miltenyi Biotec, Bergisch Gladbach, Nordrhein-Westfalen, Germany). Briefly, PBMCs (10^6^ cells/ml) were treated with different concentrations of rFg14-3-3e protein and incubated in a humidified atmosphere of 5% CO_2_ at 37 °C for 24 h. Then, PBMCs were stained with Annexin V and Propidium Iodide (PI) according to the manufacturer’s instructions. The non-treated goat PBMCs were used as a control. Annexin V-positive/PI-negative goat PBMCs were considered to be apoptotic cells and were analyzed as a percentage of the entire goat PBMCs population.

### Homology modelling of rFg14-3-3e and potential complexes with host proteins

Using protein blast [34] against the UniprotKB database [35] we retrieved the most similar human protein of rFg14-3-3e fasta protein sequence from uniprot. P62258 (YWHAE) had a Max score of 313, a Query coverage of 89% and 65% identity. Using P62258 (YWHAE), we queried the following interactions databases: Intact [36], String [37], BioPlex [38], BioGrid [39]. The resulting interacting pairs were used as input for obtaining the experimental and homology modelled structure interactions using Interactome3D [40]. All experimental and modelled interactions were used to model potential rFg14-3-3e-host interactions using FoldX [41]. The alignment of the human YWHAE with rFg14-3-3e was done using Clustal Omega [42]. Finally, a total energy analysis was done to identify stabilized and destabilized interactions. All complex structures and energy calculation files can be found in Additional file 1: Dataset S1.

### Statistical analysis

All data analyses and graphs were performed with GraphPad Premier 6.0 software package (GraphPad Prism, San Diego, California, USA). Statistical analyses were done from raw data with a one-way ANOVA, and Student’s t-tests, where applicable. Data values were expressed as means ± standard deviation (SD). Differences between tested groups were considered significant if the *P* value was ≤ 0.05, and this is indicated in the figures by asterisks (*P < 0.05; **P < 0.01; ***P < 0.001; or ****P < 0.0001). All experiments were repeated a minimum of three separate times.

## Results

### Identification, cloning and expression of rFg14-3-3e protein

To identify homologs of *F. gigantica* 14-3-3 protein in the *F. hepatica* genome, we performed a BLAST search (https://blast.ncbi.nlm.nih.gov/Blast.cgi) to screen for homologous 14-3-3 protein sequences. This analysis identified *Fh14-3-3e* gene sequence (GenBank locus: LN629113.1), which was used to design 5' and 3' primers to amplify *Fg14-3-3e*. The *Fg14-3-3e* gene was amplified and the cDNA fragment was successfully cloned into the pMD19-T cloning vector.

**Fig. 1:**
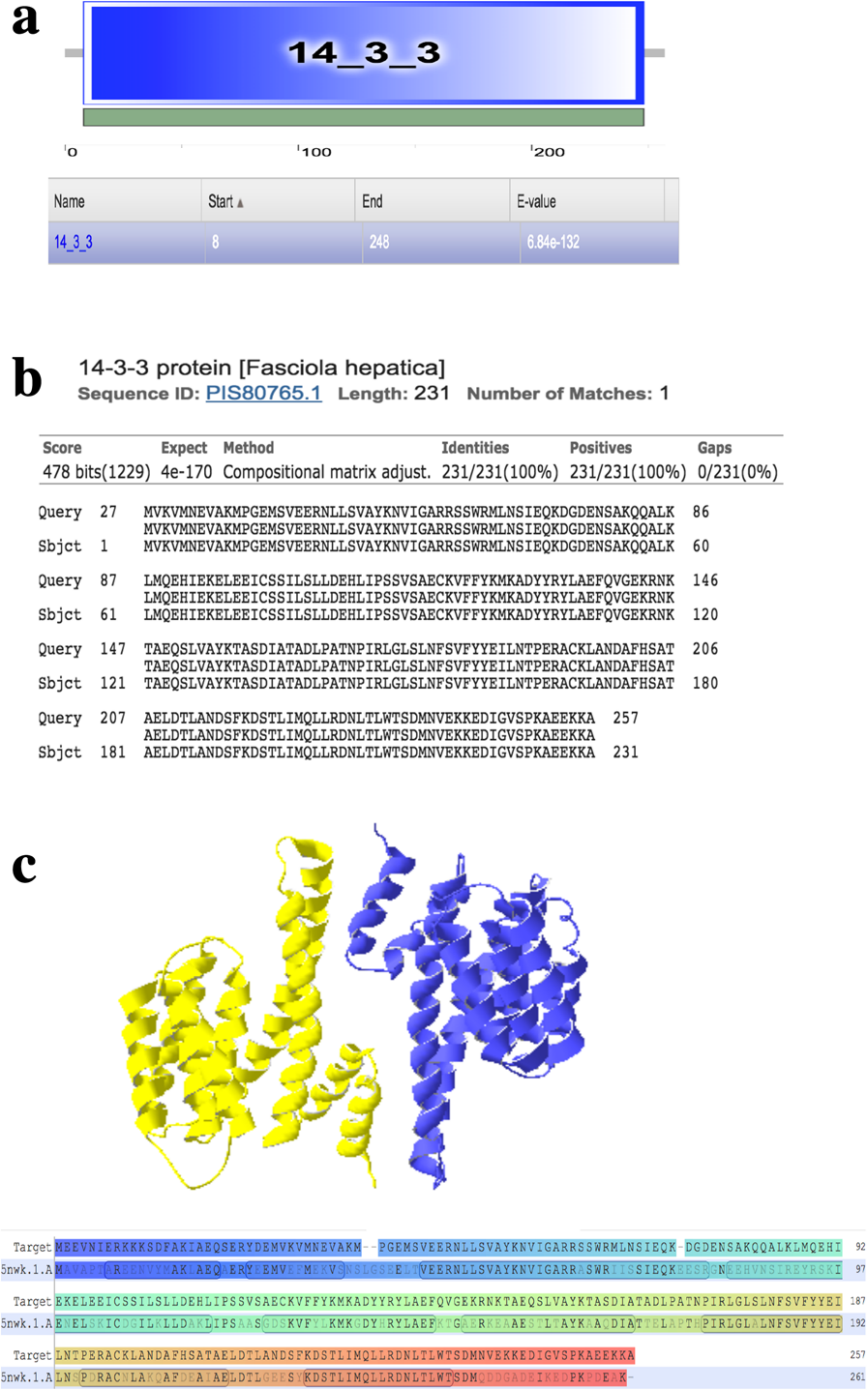
Characterization of the *Fg14-3-3e* sequence and the putative domain. **a** The predicted 14-3-3 domain involved residues 8–248, with an E-value of 6.84e-132. **b** Best Hit alignment of the Fg14-3-3e protein sequence against the NCBI non-redundant protein sequence using BLAST. The 14-3-3 protein from Fascicola hepatica (PIS80765.1) is the protein with highest similarity (100% identity, E-value 4e-170) **c** The predicted *F. gigantica Fg14-3-3e* 3D model shows 58.2% identity with the protein 14-3-3 from *Nicotiana tabacum (PDB: 5nwk)*

Nucleic acid sequencing identified positive pMD19-T-*Fg14-3-3e* clones and the obtained sequence was confirmed as *Fg14-3-3e* gene by BLAST analysis. The ORF contained 774 base-pair (bp) and encoded 257 amino acids. The SMART resource predicted the presence of a 14-3-3 domain encompassing residues 8–248, with an E-value of 6.84e-132 (Fig. 1a). The sequence similarity search showed that the 14-3-3 protein from *F. hepatica* (PIS80765.1) is the protein with highest similarity (100% identity, E-value 4e-170) (Fig. 1b). The best template found by Swissmodel to predict the *F. gigantica* 3D model was the protein 14-3-3 from *Nicotiana tabacum* (PDB: 5nwk) with 58.2% of identity (Fig. 1c). There were no N-linked glycosylation sites, signal peptides or transmembrane domains found in the deduced protein sequence.

### SDS-PAGE and Western blotting analysis

**Fig. 2:**
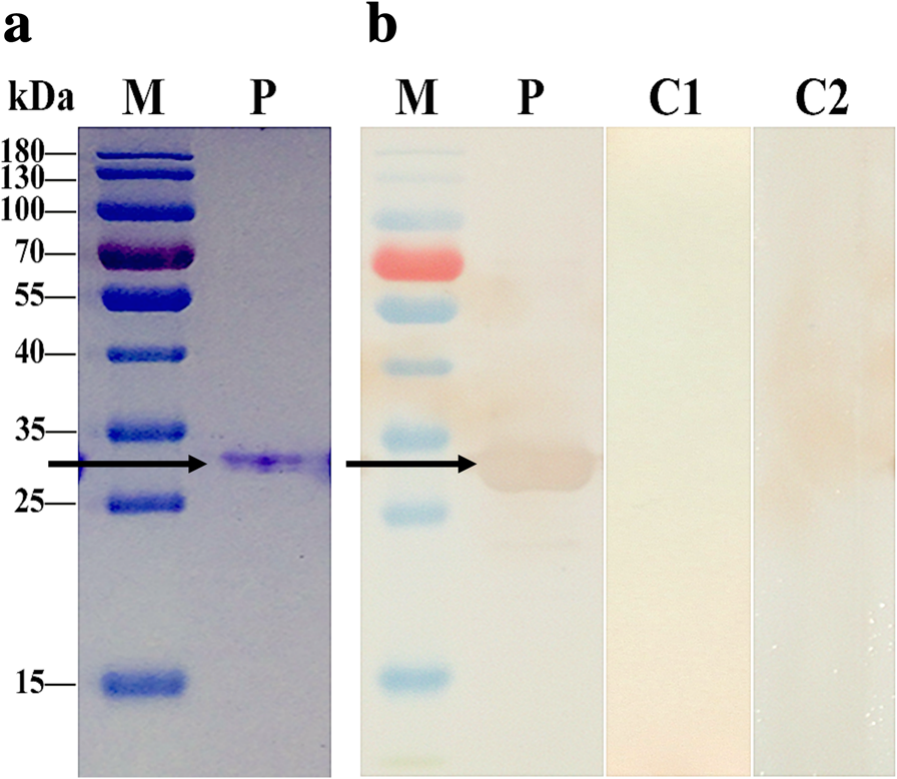
SDS-PAGE and Western blotting analyses of the extracted rFg14-3-3e protein from the culture supernatant of *P. pastoris*. **a** Proteins were resolved on 12% acrylamide gels and stained with Coomassie brilliant blue R250. Lane M: protein molecular weight marker in kDa; Lane P: purified recombinant protein, rFg14-3-3e, which appeared as a single band of approximately 29 kDa (arrow). **b** The protein of interest was run under non-reducing conditions, and visualized by immunodetection using specific antibodies and enhanced chemiluminescence. Lane M: protein molecular weight marker in kDa; Lane P: loaded with recombinant protein extracted from *P. pastoris*. Serum from *F. gigantica*-infected goats detected a single band of approximately 29 kDa (arrow); Lane C1: loaded with recombinant protein extracted from *P. pastoris* that did not react with the serum of normal, pre-immunized, goats; Lane C2: loaded with protein extracted from the supernatant of *P. pastoris* cells transformed with the pPIC9K vector without *Fg14-3-3e* gene insert, which did not react with goat serum containing anti-*F. gigantica* IgG antibodies

To verify the presence of the Fg14-3-3e protein in *F. gigantica* parasite-derived material *Fg14-3-3e* gene fragment was successfully cloned into the pPIC9K vector and the positive clones, designated as pPIC9K-*Fg14-3-3e*, were transformed into *P. pastoris* strain GS115. The recombinant protein (rFg14-3-3e) was successfully expressed in the culture supernatant of *P. pastoris*. The recombinant clones showed high expression of a novel protein of approximately 29 kDa on SDS-PAGE gels (Fig. 2a) after 72 h of induction with 1% methanol, which was absent in control samples. The specificity of this rFg14-3-3e protein was confirmed by Western blotting, which was probed with serum from goats experimentally infected with *F. gigantica*. IgG antibodies in the serum of infected goats recognized rFg14-3-3e protein monospecifically as it reacted to a single ~29 kDa band, which was absent when the Western blot was probed with serum of normal goats and in the supernatant of *P. pastoris* cells transformed with the pPIC9K vector without the Fg14-3-3e insert (Fig. 2b).

### Binding affinity of rFg14-3-3e protein to goat PBMCs

**Fig. 3:**
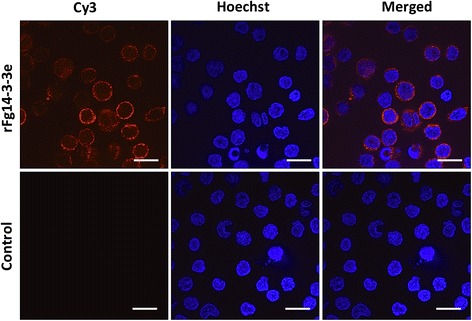
*Fasciola gigantica*-derived rFg14-3-3e protein binds to the surface of goat PBMCs. Visualization of rFg14-3-3e protein attachment to goat PBMCs surface was carried out by incubation of goat PBMCs treated or untreated with rFg14-3-3e protein with rat anti-rFg14-3-3e primary antibody. Hoechst (blue) and Cy3-conjugated secondary antibody (red) were used to stain host cell nuclei and rFg14-3-3e protein, respectively. Surface staining was detected in rFg14-3-3e-treated cells. No staining was detectable in untreated cells. *Scale-bars*: 10 μm

Binding of rFg14-3-3e protein to the surface of goat PBMCs was assessed by immunofluorescent staining. Tagging rFg14-3-3e-treated goat PBMCs with specific rat anti-rFg14-3-3e antibodies, as indicated by the concentration of red Cy3 dye, showed successful binding of rFg14-3-3e to the cell surface (Fig. 3), whereas, no fluorescence was observed in the non-treated cells.

### rFg14-3-3e protein modulated cytokine production by goat PBMCs

**Fig. 4:**
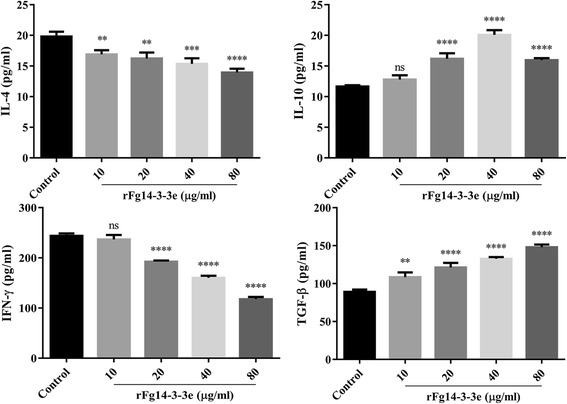
rFg14-3-3e protein induced polarized patterns of cytokine secretion. Goat PBMCs were incubated for 24 h in the presence or absence of serial concentrations of rFg14-3-3e protein. The levels of cytokine concentration in the supernatant of cultured goat PBMCs was quantified by ELISA. Graphs represent means ± standard deviations of data from 3 independent biological replicates. Asterisks indicate statistical significance between treated and untreated control goat PBMCs (*P < 0.05; **P < 0.01; ***P < 0.001; ****P < 0.0001; ns, non-significant)

To gain insight into how rFg14-3-3e protein modulates cytokine profiles of goat PBMCs, the levels of four cytokines, IL-4, IL-10, IFN-γ, and TGF-β, were determined. As shown in Fig. 4, when goat PBMCs were treated with serial concentrations of rFg14-3-3e protein, the production of IL-4 (10 μg/ml: ANOVA, *F*_(4, 10)_ = 21.77, *P* = 0.0089; 20 μg/ml: ANOVA, *F*_(4, 10)_ = 21.77, *P* = 0.0021; 40 μg/ml: ANOVA, *F*_(4, 10)_ = 21.77, *P* = 0.0004; 80 μg/ml: ANOVA, *F*_(4, 10)_ = 21.77, *P* < 0.0001) and IFN-γ (10 μg/ml: ANOVA, *F*_(4, 10)_ = 292.5, *P* = 0.5123; 20 μg/ml: ANOVA, *F*_(4, 10)_ = 292.5, *P* < 0.0001; 40 μg/ml: ANOVA, *F*_(4, 10)_ = 292.5, *P* < 0.0001; 80 μg/ml: ANOVA, *F*_(4, 10)_ = 292.5, *P* < 0.0001) were significantly decreased compared to the control goat PBMCs. On the other hand, the production of IL-10 (10 μg/ml: ANOVA, *F*_(4, 10)_ = 79.19, *P* = 0.2803; 20 μg/ml: ANOVA, *F*_(4, 10)_ = 79.19, *P* < 0.0001; 40 μg/ml: ANOVA, *F*_(4, 10)_ = 79.19, *P* < 0.0001; 80 μg/ml: ANOVA, *F*_(4, 10)_ = 79.19, *P* < 0.0001) and TGF-β (10 μg/ml: ANOVA, *F*_(4, 10)_ = 74.03, *P* = 0.0027; 20 μg/ml: ANOVA, *F*_(4, 10)_ = 74.03, *P* < 0.0001; 40 μg/ml: ANOVA, *F*_(4, 10)_ = 74.03, *P* < 0.0001; 80 μg/ml: ANOVA, *F*_(4, 10)_ = 74.03, *P* < 0.0001) were increased.

### rFg14-3-3e protein inhibited cell proliferation

**Fig. 5:**
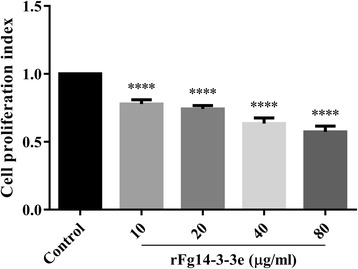
rFg14-3-3e protein inhibited goat PBMCs proliferation. Goat PBMCs were sham-treated with control buffer or with serial concentrations of rFg14-3-3e protein and incubated for 72 h at 37 °C at 5% CO_2_. Proliferation of cells was determined using CCK-8 assay. Results indicate that rFg14-3-3e protein significantly inhibited goat PBMCs proliferation. Graphs represent means ± standard deviations of data from 3 independent biological replicates. Asterisks indicate significance difference between treated cells and control cells (****P < 0.0001)

To determine whether rFg14-3-3e protein has any influence on the proliferation of goat PBMCs, CCK-8 assay was used. As shown in Fig. 5, the proliferation of goat PBMCs was significantly inhibited following treatment with rFg14-3-3e protein, at all tested protein concentrations (10 μg/ml: ANOVA, *F*_(4, 25)_ = 157.2, *P* < 0.0001; 20 μg/ml: ANOVA, *F*_(4, 25)_ = 157.2, *P* < 0.0001; 40 μg/ml: ANOVA, *F*_(4, 25)_ = 157.2, *P* < 0.0001; 80 μg/ml: ANOVA, *F*_(4, 25)_ = 157.2, *P* < 0.0001). The anti-proliferative effect of rFg14-3-3e protein was concentration-dependent.

### rFg14-3-3e protein did not stimulate cell migration

**Fig. 6:**
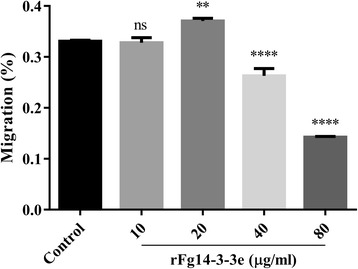
rFg14-3-3e protein suppressed goat PBMCs migration. Goat PBMCs were sham-treated with control buffer or with serial concentrations of rFg14-3-3e protein, then the cell migration percentage (%) was determined. Graphs represent means ± standard deviations of data from 3 independent biological replicates. The asterisks indicate significant difference between treated and sham-treated control cells (**P < 0.001; ****P < 0.0001; ns, non-significant)

Here, we tested whether rFg14-3-3e protein stimulates the migration of goat PBMCs using 6.5 mm Transwell® with 8.0 μm Pore Polyester Membrane Inserts. As shown in Fig. 6, 40 μg/ml and 80 μg/ml of rFg14-3-3e protein significantly suppressed the migration of goat PBMCs compared with the controls, but the 10 μg/ml and 20 μg/ml did not (10 μg/ml: ANOVA, *F*_(4, 10)_ = 361.4, *P* = 0.9902; 20 μg/ml: ANOVA, *F*_(4, 10)_ = 361.4, *P* = 0.0011; 40 μg/ml: ANOVA, *F*_(4, 10)_ = 361.4, *P* < 0.0001; 80 μg/ml: ANOVA, *F*_(4, 10)_ = 361.4, *P* < 0.0001).

### Nitric oxide production

**Fig. 7:**
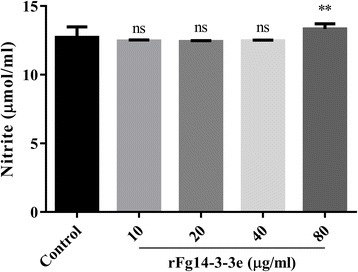
Effects of rFg14-3-3e protein on intracellular NO production. Goat PBMCs were sham-treated with control buffer or with serial concentrations of rFg14-3-3e protein and maintained at 37 °C. NO concentration in the goat PBMCs was measured by Griess assay. Graphs represent means ± standard deviations of data from 3 independent biological replicates. Asterisks indicate significant difference between treated and non-treated control cells (**P < 0.01; ns, non-significant). The inhibitory effect was only statistically significant at the highest concentration (80 μm/ml)

As shown in Fig. 7, NO release was slightly increased by goat PBMCs in the presence of rFg14-3-3e protein at 80 μg/ml dose compared to the control, but not at 10 μg/ml, 20 μg/ml or 40 μg/ml (10 μg/ml: ANOVA, *F*_(4, 40)_ = 8.695, *P* = 0.4307; 20 μg/ml: ANOVA, *F*_(4, 40)_ = 8.695, *P* = 0.3221; 40 μg/ml: ANOVA, *F*_(4, 40)_ = 8.695, *P* = 0.5012; 80 μg/ml: ANOVA, *F*_(4, 40)_ = 8.695, *P* = 0.0063). These results suggest that increased NO production may become apparent only with exposure to a larg rFg14-3-3e protein concentration.

### rFg14-3-3e protein reduced cell phagocytosis

**Fig. 8:**
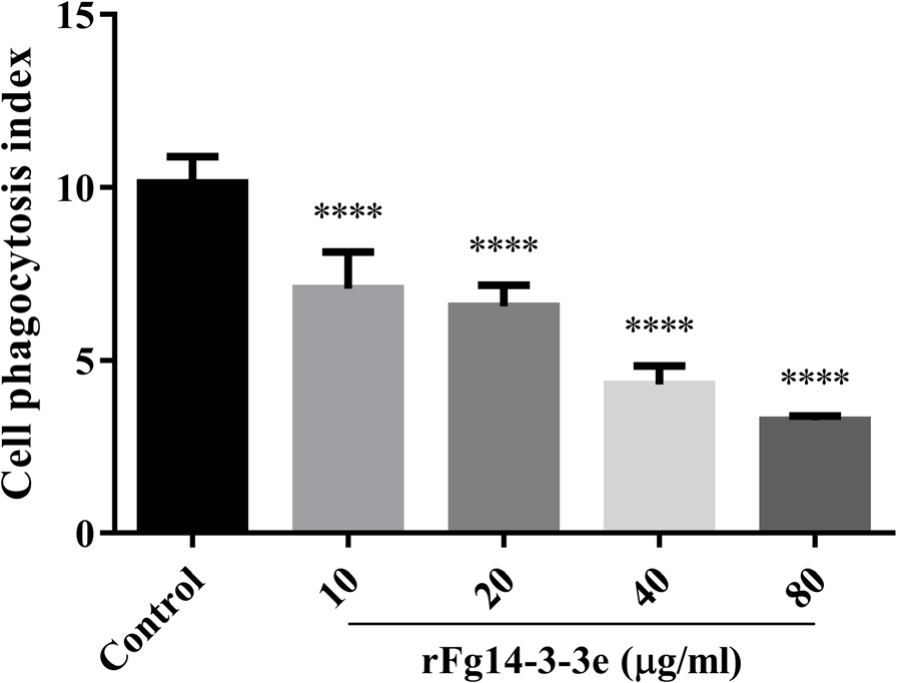
rFg14-3-3e protein suppressed goat cell phagocytosis. rFg14-3-3e protein inhibited the phagocytic ability of monocytes as indicated by the reduction in FITC-dextran uptake in a dose-dependent manner. Graphs represent means ± standard deviations of data from 3 independent biological replicates. Significance was set at ****P < 0.0001 compared to sham-treated monocytes

The phagocytic ability of goat monocytes was examined via assessment of FITC-dextran uptake. Results revealed that treatment with rFg14-3-3e protein at all tested protein concentrations suppressed goat monocyte phagocytosis (10 μg/ml: ANOVA, *F*_(4, 25)_ = 59.52, *P* < 0.0001; 20 μg/ml: ANOVA, *F*_(4, 25)_ = 59.52, *P* < 0.0001; 40 μg/ml: ANOVA, *F*_(4, 25)_ = 59.52, *P* < 0.0001; 80 μg/ml: ANOVA, *F*_(4, 25)_ = 59.52, *P* < 0.0001) (Fig. 8). rFg14-3-3e protein reduced FITC-dextran phagocytosis by approximately 32%, 42%, 65% and 70% at 10 μg/ml, 20 μg/ml, 40 μg/ml and 80 μg/ml, respectively.

### rFg14-3-3e protein stimulated cell apoptosis

**Fig. 9:**
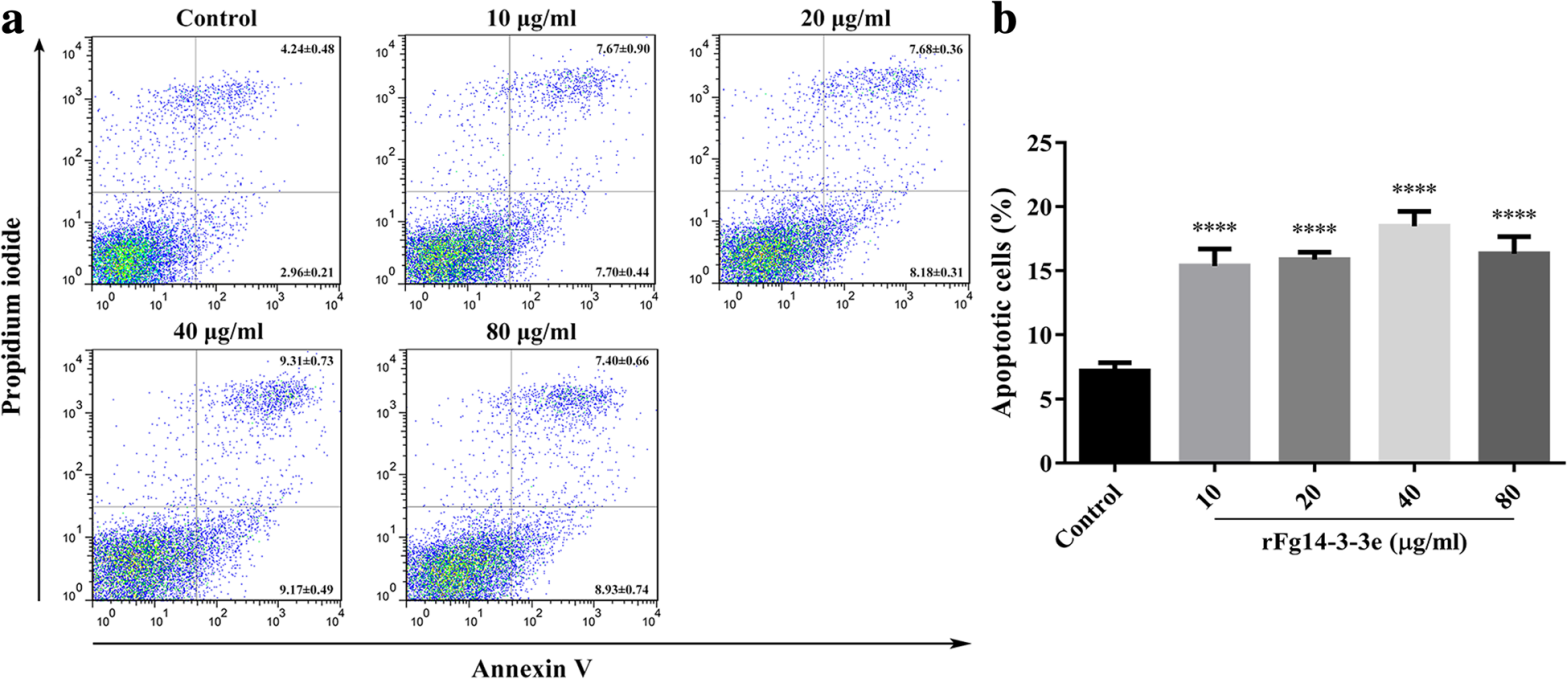
rFg14-3-3e protein induced apoptosis in goat PBMCs. Apoptotic cells were determined by Annexin V/PI staining and flow cytometry analysis. **a** Dot plot showing death of goat PBMCs in response to exposure to rFg14-3-3e protein. **b** Apoptotic cells (Annexin V+/PI-) were plotted and compared with percentage of cell population. Graphs represent means ± standard deviations of data from 3 independent biological replicates. The asterisks indicate significant differences between treated and untreated control goat PBMCs (****P < 0.0001)

To explore whether rFg14-3-3e protein has an apoptotic effect on goat PBMCs, anenxin V-FITC/PI double staining apoptosis assay was used. The results showed that rFg14-3-3e protein significantly induced, in a dose-dependent manner, apoptosis in goat PBMCs at all tested concentrations compared to untreated control goat PBMCs (10 μg/ml: ANOVA, *F*_(4, 37)_ = 104.4, *P* < 0.0001; 20 μg/ml: ANOVA, *F*_(4, 37)_ = 104.4, *P* < 0.0001; 40 μg/ml: ANOVA, *F*_(4, 37)_ = 104.4, *P* < 0.0001; 80 μg/ml: ANOVA, *F*_(4, 37)_ = 104.4, *P* < 0.0001) (Fig. 9).

### Modelled protein interactions suggest host interacting partners of the rFg14-3-3e protein

**Fig. 10:**
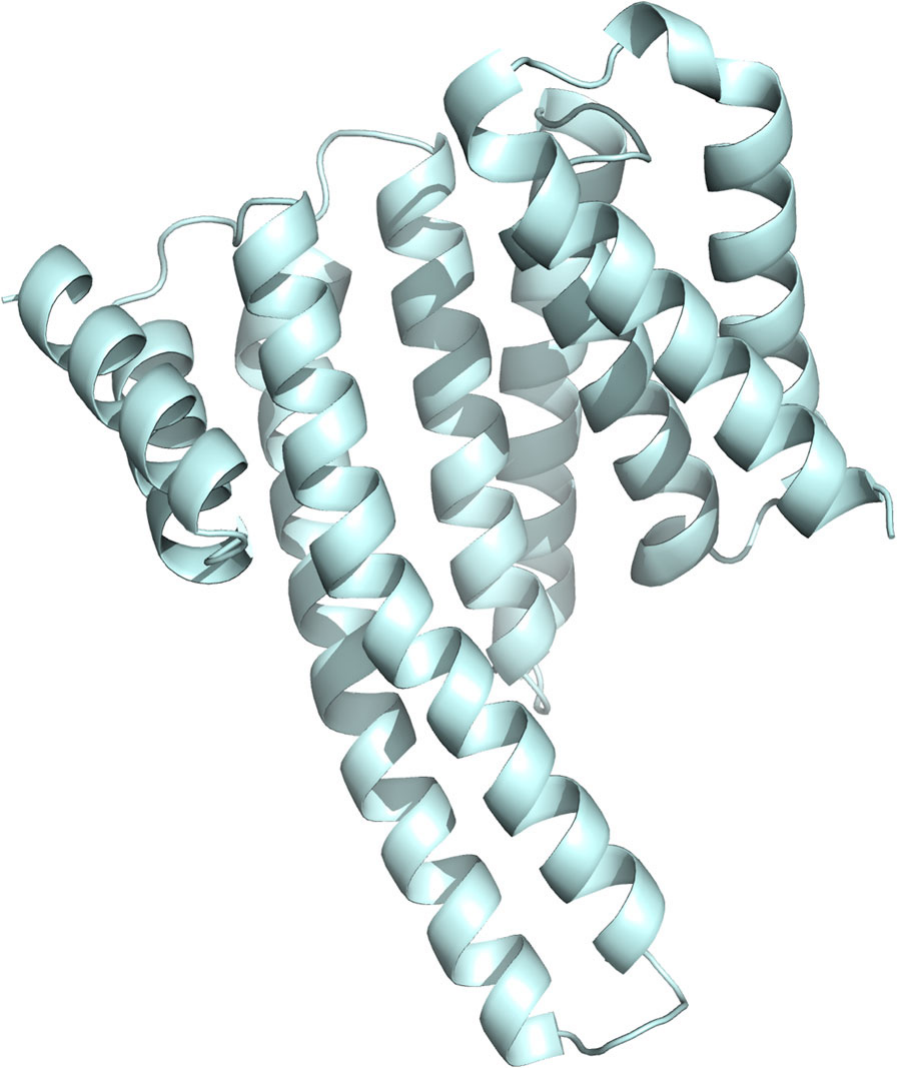
3D visualization of the rFg14-3-3e molecule. Molecular homology modelling using FoldX was used to predict a three-dimensional representation of the rFg14-3-3e protein. The predicted rFg14-3-3e 3D model shows 65% identity with the human 14-3-3 protein YWHAE

We performed homology modelling using the human 14-3-3 protein YWHAE, which had 65% identity to the rFg14-3-3e using FoldX (Fig. 10). We then used Interactome3D [40] to model protein complexes involving YWHAE and its human interactors. Interactome3D retrieved 52 complex models between P62258 and other 18 human proteins. Multiple models were made per complex based on different complex templates that were available in the PDB [43]. We then used FoldX [41] to model the interaction between the rFg14-3-3e and these human proteins, and to calculate the changes in free energy for these complexes. Additional file 2: Table S1 shows the list of interactions with its respective total energy for the human YWHAE protein and the modelled rFg14-3-3e protein, and the difference of the previous values. More negative energy values for the rFg14-3-3e compared to the human YWHAE indicate increased complex stability, and may suggest potential interactors of the parasite protein in the host. Positive energy values indicate unstable complexes and thus unfavorable interactions. We observed negative values for 13 out of the 18 potentially interacting proteins. Enrichment analysis of the interacting proteins using Funcassociate 3.0 [44] identified Gene Ontology (GO) terms related to apoptosis, protein binding, locomotion, hippo signalling and leukocyte, and lymphocyte differentiation (Additional file 3: Table S2).

## Discussion

Here, *Fg14-3-3e* gene was successfully cloned, expressed and the recombinant protein (rFg14-3-3e) was obtained. Sequence analysis of this recombinant protein indicated that rFg14-3-3e protein is a member of the 14-3-3 family, which mediates metabolism and signal transduction networks through binding to hundreds of other protein partners [15, 18–20]. The 14-3-3 protein has been studied in other parasitic helminths [45] and Fh14-3-3e protein was able to bind to goat PBMCs *in vivo* [46].

The aim of the present study was to investigate the immunomodulatory effects of rFg14-3-3e protein on goat innate immunity. To achieve this, we cloned and expressed Fg14-3-3 protein in a heterologous system. Our results demonstrated the successful expression of rFg14-3-3e with a molecular mass of approximately 29kDa, which was validated by Western blotting using sera from *F. gigantica*-experimentally-infected goats. This rFg14-3-3e protein also reacted with the rat anti-rFg14-3-3e antibodies as indicated by specific binding of rFg14-3-3e protein to the surface of goat PBMCs, suggesting that rFg14-3-3e protein might be involved in interaction with cellular elements of the innate immune system during *F. gigantica* infection in goats. This immunogenicity of rFg14-3-3e protein makes it a good candidate for the development of a diagnostic assay for *F. gigantica* infection.

Our data also showed that goat PBMCs exposed to rFg14-3-3e protein change their cytokine production profile and undergo apoptosis *in vitro*. In particular, rFg14-3-3e-induced the production of IL-10 and TGF-β, while reduced the production of IL-4 and IFN-γ, suggesting that rFg14-3-3e protein has polarized the goat PBMCs’ immune cytokine pattern. Earlier studies indicated that apoptotic cells may be involved in suppressing inflammatory responses by inducing the anti-inflammatory cytokines, IL-10 [47, 48] and TGF-β [49, 50]. IL-10 and TGF-β secreted by allergen-specific type 1 regulatory (Tr1) cells were found to cause immunosuppression *via* inhibition of T-cell activation and differentiation [51]. Therefore, both IL-10 and TGF-β cytokines released from apoptotic goat PBMCs are likely to modulate immune-inflammatory responses during *F. gigantica* infection.

Our observation that rFg14-3-3e protein suppresses immune response is in line with what we described previously during *F. gigantica* infection in buffaloes. An ‘early’ TGF-β-associated immuno-suppressive response together with upregulation of liver IL-10 mRNA expression [52] and increased level of serum IL-10 cytokine [53] have been observed in buffaloes during early *F. gigantica* infection. In another study, we reported downregulation of MHC-II related genes and suppression of the host pro-inflammatory (Th1) immune response during early *F. gigantica* infection in buffalo liver [54]. This observation extends to *F. hepatica*, where immunosuppression, mediated by IL-4 and IL-10, was reported in experimentally infected rats during the early stage of liver penetration [55]. In the context of *F. gigantica* infection, suppression of immune response may have beneficial effects in terms of promoting parasite survival, while minimizing the inflammatory pathological damage in the host.

We subsequently showed that rFg14-3-3e protein significantly inhibited the proliferation and migration of treated goat PBMCs compared to untreated cells. Proliferation and migration of host immune cells and production of cytokines are essential for the control of parasite infection [56, 57]. Also, we found that rFg14-3-3e protein inhibits the phagocytic activity of monocytes, and increases the NO release only at a high concentration. The abomasal nematode *Haemonchus contortus* was also found to inhibit phagocytosis, production of NO and free radicals of host monocytes [58]. These previous results and our findings indicate that similar to14-3-3 protein of other parasites, rFg14-3-3e protein can actively suppress host monocyte phagocytosis, and inhibits the proliferation and migration of treated goat PBMCs to promote the survival of the parasite.

Next, we detected a significantly high degree of rFg14-3-3e-induced apoptosis in goat PBMCs. 14-3-3 proteins are key regulators of protein kinase signaling cascades and can play many roles in apoptosis, signal transduction, and cell cycle regulation [59, 60]. Induction of apoptosis has also been reported in the peritoneal leucocytes of sheep experimentally infected with *F. hepatica* during early infection in an effort to support the juvenile flukes’ survival during the peritoneal migration to the liver [61]. Transcriptomic analysis showed that *F. hepatica* may attenuate the inflammatory response through induction of apoptosis in goat PBMCs [62]. Therefore, it can be assumed that reduced proliferation and increased apoptosis of goat PBMCs in response to exposure to rFg14-3-3e may be survival mechanisms used by *F. gigantica* to evade host immune defence mechanisms. Both the death receptor pathway (extrinsic) and the mitochondrial pathway (intrinsic) seemed to contribute to *F. hepatica*-induced apoptosis in goat PBMCs [62]. However, apoptotic mechanisms vary in response to different parasite species or host cell types. Therefore, further studies are required to investigate the process by which rFg14-3-3e protein causes death of goat PBMCs.

The knowledge of the 3D structure of rFg14-3-3e protein can help in the understanding of its function and knowledge of the extent of molecular interaction between this protein and other host proteins may help in future design of novel molecules useful to modulate its activity. In our work, we predicted the three-dimensional model of the homo-dimer using homology modelling (Swissmodel). This prediction strategy identified the protein 14-3-3 from *Nicotiana tabacum* (PDB: 5nwk) as the best template to predict the *F. gigantica* 3D model with 58.2% identity. We were also interested in studying potential host protein interactors with rFg14-3-3e protein. Therefore, we searched the UniprotKB database in order to identify a human protein that has the highest similarity to rFg14-3-3e fasta protein sequence. This analysis revealed 65% identity between rFg14-3-3e and human P62258 (YWHAE). Human YWHAE gene codes for 14-3-3 protein epsilon, which is implicated in the regulation of a large spectrum of signaling pathways. YWHAE was used as input to identify potential interactions of YWHAE protein with other human proteins in various interaction databases. The interacting pairs were then used to obtain experimental and homology modelled structure interactions using Interactome3D. All identified experimental and modelled interactions were used to model potential rFg14-3-3e-host interactions using FoldX. The majority (13 of the 18) of the potentially interacting proteins of rFg14-3-3e compared to the human YWHAE were negative, suggesting more interconnectivity of the parasite protein with the host protein. We also, performed GO enrichment analysis, which identified terms related to apoptosis, protein binding, locomotion, hippo signaling, and leukocyte and lymphocyte differentiation, all critical pieces of the immunopathogenesis of *F. gigantica* infection. These findings indicate that results obtained by *in silico* analysis support the reliability of the experimental findings and demonstrate that rFg14-3-3e is involved in a number of immunoregulatory activities.

## Conclusions

The results obtained in this study showed clear modulatory effects of rFg14-3-3e protein on multiple functions of goat PBMCs *in vitro*. rFg14-3-3e protein interacted with sera from goats experimentally infected with *F. gigantica* and with rat anti-rFg14-3-3e antibody. Also, rFg14-3-3e protein stimulated the concurrent production of IL-10 and TGF-β, and promoted apoptosis of goat PBMCs. In addition, rFg14-3-3e protein reduced the production of IL-4 and IFN-γ, inhibited proliferation and migration of goat PBMCs, and suppressed monocyte phagocytic ability. Results obtained from experimental studies were supported by homology modelling and GO enrichment analysis. These findings indicate that *F. gigantica* rFg14-3-3e protein, may play important roles in the interaction of *F. gigantica* with goat PBMCs. Our data may lead to a better understanding of *F. gigantica* interactions with goat PBMCs and fasciolosis pathogenesis. These results may be relevant for the identification of potential novel immunomodulatory therapies and preventative strategies, since antibodies or chemical inhibitors against rFg14-3-3e protein can interfere with important mechanisms in parasite survival. Further studies are warranted to determine the immunomodulatory role of rFg14-3-3e protein in the context of *F. gigantica* infection in goats.

## Additional files

Additional file 1: **Dataset S1.** Structure files, FoldX configuration files and associated energy calculations for complexes between YWAHE and rFg14-3-3, and the 18 human protein interactors of YWAHE. (TGZ 5273 kb)

Additional file 2: **Table S1.** The list of interactions of rFg14-3-3e protein with its respective total energy for **a** the human YWHAE protein, **b** the modelled rFg14-3-3e protein and the difference of the previous values. (XLSX 15 kb)

Additional file 3: **Table S2.** Enrichment analysis results of the interacting proteins using Funcassociate 3.0 [44]. (XLSM 18 kb)

## Abbreviations

BSA: Bovine serum albumin
cDNA: Complementary deoxyribonucleic acid
DAB: 3,3′-diaminobenzidine
ELISA: Enzyme linked immunosorbent assay
ESP: Excretory and secretory product
FBS: Fetal bovine serum
FITC: Fluorescein isothiocyanate
GO: Gene Ontology
IFA: Immunofluorescence assay
IFN-γ: Interferon gamma
IgG: Immunoglobulin G
IL-10: Interleukin 10
IL-4: Interleukin 4
ITS: Internal transcribed spacer
LC-MS/MS: Liquid chromatography-tandem mass spectrometry
MFI: Median fluorescence intensity
MHC: Major histocompatibility complex
NO: Nitric oxide
ORF: Open reading frame
PBMC: Peripheral blood mononuclear cell
PBS: Phosphate-buffered solution
PI: Propidium iodide
PS: Penicillin-streptomycin
RNA: Ribonucleic acid
RT-PCR: Reverse transcription polymerase chain reaction
SD: Sprague Dawley
SD: Standard deviation
SDS-PAGE: Sodium dodecyl sulfate polyacrylamide gel electrophoresis
SMART: Simple modular architecture research tool
TBST: Tris-buffered saline containing 0.1% tween-20
TGF-β: Transforming growth factor beta
Treg: T regulatory cells
